# Time series decomposition into dyslipidemia prevalence among urban Chinese population: secular and seasonal trends

**DOI:** 10.1186/s12944-021-01541-6

**Published:** 2021-09-22

**Authors:** Jiahui Lao, Yafei Liu, Yang Yang, Peng Peng, Feifei Ma, Shuang Ji, Yujiao Chen, Fang Tang

**Affiliations:** 1 Department of Endocrinology and Metabology, The First Affiliated Hospital of Shandong First Medical University & Shandong Provincial Qianfoshan Hospital, Jinan, China; 2 Center for Big Data Research in Health and Medicine, The First Affiliated Hospital of Shandong First Medical University & Shandong Provincial Qianfoshan Hospital, Jingshi Road 16766 250014 Jinan, China; 3 Shandong Provincial Qianfoshan Hospital, Cheeloo College of Medicine, Shandong University, Jinan, China; 4 School of Public Health, Weifang Medical University, Weifang, China

**Keywords:** Dyslipidemia, Time series decomposition, Secular trends, Seasonal trends, Hypercholesterolemia, Hypertriglyceridemia, Mixed hyperlipidemia, HDL-C hypolipidemia

## Abstract

**Background:**

Previous epidemiological studies have indicated the seasonal variability of serum lipid levels. However, little research has explicitly examined the separate secular and seasonal trends of dyslipidemia. The present study aimed to identify secular and seasonal trends for the prevalence of dyslipidemia and the 4 clinical classifications among the urban Chinese population by time series decomposition.

**Methods:**

A total of 306,335 participants with metabolic-related indicators from January 2011 to December 2017 were recruited based on routine health check-up systems. Multivariate direct standardization was used to eliminate uneven distributions of the age, sex, and BMI of participants over time. Seasonal and trend decomposition using LOESS (STL decomposition) was performed to break dyslipidemia prevalence down into trend component, seasonal component and remainder component.

**Results:**

A total of 21.52 % of participants were diagnosed with dyslipidemia, and significant differences in dyslipidemia and the 4 clinical classifications were observed by sex (*P* <0.001). The secular trends of dyslipidemia prevalence fluctuated in 2011–2017 with the lowest point in September 2016. The dyslipidemia prevalence from January to March and May to July was higher than the annual average (λ = 1.00, 1.16, 1.06, 1.01, 1.02, 1.03), with the highest point in February. Different seasonal trends were observed among the 4 clinical classifications. Compared to females, a higher point was observed among males in February, which was similar to participants aged < 55 years (vs. ≥ 55 years) and participants with a BMI ≤ 23.9 (vs. BMI > 23.9).

**Conclusions:**

There were significant secular and seasonal features for dyslipidemia prevalence among the urban Chinese population. Different seasonal trends were found in the 4 clinical classifications of dyslipidemia. Precautionary measures should be implemented to control elevated dyslipidemia prevalence in specific seasons, especially in the winter and during traditional holidays.

**Supplementary Information:**

The online version contains supplementary material available at 10.1186/s12944-021-01541-6.

## Background

Dyslipidemia is characterized by increased total cholesterol (TC), low-density lipoprotein cholesterol (LDL-C), and triglyceride (TG) levels and/or reduced high-density lipoprotein cholesterol (HDL-C) levels [1]. Previous studies have shown that dyslipidemia could increase the risk of cardiac-cerebral vascular diseases, such as coronary artery disease (CAD) and stroke [2, 3]. Dyslipidemia causes over 50 % of the CAD patients worldwide [4]. In China, the prevalence of dyslipidemia of adults is approximately 34.0 % [5]. With the urbanization and improvement of the Chinese population’s quality of life, the Chinese dyslipidemia prevalence is constantly increasing, especially in urban areas [6, 7].

Epidemiological research has found seasonal variability of serum lipid and cardiac-cerebral vascular diseases. Previous studies suggested that TC levels were higher in cold seasons than hot seasons [8]. Peaks in winter were also found among cardiovascular diseases [9]. Considering the well-known relationship between serum lipid levels and cardiac-cerebral vascular diseases, seasonal variability in serum lipid levels might result in different incidences of cardiac-cerebral vascular diseases among seasons.

Abnormal serum lipid levels cause increased hazards of CAD and other cardiac-cerebral vascular diseases [10, 11]. The identification of seasonal trends of dyslipidemia prevalence is crucial to the prevention and control of related diseases. A few studies based on time series have explored seasonal trends of dyslipidemia [12]. To our knowledge, no study has separated secular trends and remainder from seasonal trends of dyslipidemia, which might help clarify seasonal variations in dyslipidemia prevalence. Moreover, most of the studies did not have sufficiently long research periods or sufficient participants. Therefore, the present study, based on the routine health check-up system, collected large-scale urban population health information which minimized potential recall and response biases [13], and first aimed to identify secular and seasonal trends of dyslipidemia prevalence among urban Chinese adults by time-series decomposition and then assess the different trends for the 4 clinical classifications and subgroups.

## Materials and methods

### Participants

This study included 306,335 participants aged 20 years or older who were attending routine health check-up program at the Center for Health Management of Shandong Provincial Qianfoshan Hospital with serum lipids measurements from January 2011 to December 2017.

Eligible participants were (1) aged ≥ 20 years and (2) with health records for TC, TG, HDL-C and LDL-C measurements, and age, sex, and body mass index (BMI) information. Participants were excluded if they had no exact check-up time that was recorded.

### Measurements

According to the standard clinical and laboratory protocol, serum lipid levels were measured by the Roche Cobase c701 testing system. TC and TG were measured by enzyme colorimetry. HDL-C and LDL-C were measured by the direct catalase scavenging method and selective clearance method, respectively.

### Definition of dyslipidemia

Based on the “2016 Chinese guidelines for the management of dyslipidemia in adults” [14], people will be diagnosed with dyslipidemia when: TC ≥ 6.2 mmol/L (240 mg/dl) or TG ≥ 2.3 mmol/L (200 mg/dl) or HDL-C < 1.0 mmol/L (40 mg/dl) or LDL-C ≥ 4.1 mmol/L (160 mg/dl). Dyslipidemia was further classified into 4 clinical classifications [14]: hypercholesterolemia (TC ≥ 6.2 mmol/L or 240 mg/dl), hypertriglyceridemia (TG ≥ 2.3 mmol/L or 200 mg/dl), mixed hyperlipidemia (TC ≥ 6.2 mmol/L or 240 mg/dl and TG ≥ 2.3 mmol/L or 200 mg/dl), HDL-C hypolipidemia (HDL-C < 1.0 mmol/L or 40 mg/dl).

### Statistical analysis

Before time series decomposition, chi-square tests were performed to analyze the difference in the prevalence of dyslipidemia and the 4 clinical classifications between males and females.

Considering the influence of age, sex, and BMI on dyslipidemia and uneven distribution of age, sex, and BMI of participants over time, adjusted prevalence was calculated by multivariate direct standardization, using the participants in the year 2011 of this study as the standard population.

The method of direct standardization was used as shown below:

$$p{\prime }=\frac{\sum {N}_{\text{i}}{p}_{\text{i}}}{N}$$

where $p{\prime }$ is the standardized prevalence, ${N}_{\text{i}}$ is the number of standard populations of each group, ${p}_{\text{i}}$ is the unstandardized prevalence of each group, and $N$ is the total number of standard populations.

A time series can be broken down into trend-cycle component (also called “trend”, ${T}_{\text{t}}$), seasonal component (${S}_{\text{t}}$) and remainder component (${R}_{\text{t}}$) [15]. In additive decomposition, the equation is ${y}_{\text{t}}={S}_{\text{t}}+{T}_{\text{t}}+{R}_{\text{t}}$. In the equation, ${y}_{\text{t}}$ is the time series data. Similarly, a multiplicative decomposition is ${y}_{\text{t}}={S}_{\text{t}}\text{*}{T}_{\text{t}}\text{*}{R}_{\text{t}}$.

After calculating the adjusted dyslipidemia prevalence for each month, the method of seasonal and trend decomposition using LOESS (STL decomposition) was used to break the prevalence down into trend component, seasonal component and remainder component [16]. LOESS (locally weighted regression) is a nonparametric method for local regression analysis for estimating nonlinear relationships. Inner loop and outer loop are included in the process of STL decomposition. The inner loop was used for trend fitting and cycle component calculation. The outer loop was used to regulate the robustness weight. Compared with classical decomposition methods, STL decomposition is more robust in reducing the effect of unusual observations on the estimates of the trend and seasonal components.

STL decomposition only provides facilities for additive decompositions. However, multiplicative decomposition was more appropriate for dyslipidemia prevalence in this study. Therefore, logarithmic transformation was conducted on the time series before STL decomposition. After decomposition, an exponential transformation was applied for the trend component, seasonal component and remainder component. When λ of the seasonal trends was greater than 1, it meant that the prevalence was higher than the annual average.

Subgroup analysis was performed by the same methods described above to identify the different secular and seasonal trends between participants of different characteristics.

Although multiplicative decomposition was considered more appropriate in this study, there was no definite quantitative determining criterion between the usage of multiplicative and additive decomposition. Therefore, sensitivity analysis was conducted for dyslipidemia prevalence by using STL decomposition without logarithmic transformation and exponential transformation, which is an additive decomposition approach.

R software (version 3.3.1) was used to conduct data analysis. The ‘‘stl()’’ function was used to develop STL decomposition. The level of statistical significance was 0.05 (two-sided).

## Results

### Distribution and prevalence

In total, 306,335 participants were included in the present study, comprising 185,490 males (accounting for 60.55 %, mean age = 46.2, standard deviation (SD) = 14.5) and 120,845 females (accounting for 39.45 %, mean age = 44.8, SD = 13.9). A total of 21.52 % of participants were diagnosed with dyslipidemia. The prevalence was 26.05 % for males and 14.58 % for females. From 2011 to 2017, the dyslipidemia prevalence of all participants, males, and females was highest in 2013 and equal to 25.84 %, 30.67 %, and 17.59 %, respectively.

As shown in Table 1, males had a higher prevalence of dyslipidemia, hypertriglyceridemia, mixed hyperlipidemia and HDL-C hypolipidemia than females, but a lower hypercholesterolemia prevalence than females (all *P* < 0.001).

**Table 1:** Prevalence of dyslipidemia and 4 clinical classifications during 2011–2017

Clinical classification	All (%)	Males (%)	Females (%)	χ^2^	*P*
Dyslipidemia	21.52	26.05	14.58	3757.23	<0.001
Hypercholesterolemia	7.06	6.24	8.32	416.09	<0.001
Hypertriglyceridemia	11.43	15.28	5.53	5573.25	<0.001
HDL-C hypolipidemia	6.38	9.12	2.17	5280.69	<0.001
Mixed hyperlipidemia	1.81	2.09	1.36	212.15	<0.001

### Secular and seasonal trends of dyslipidemia prevalence

**Fig. 1:**
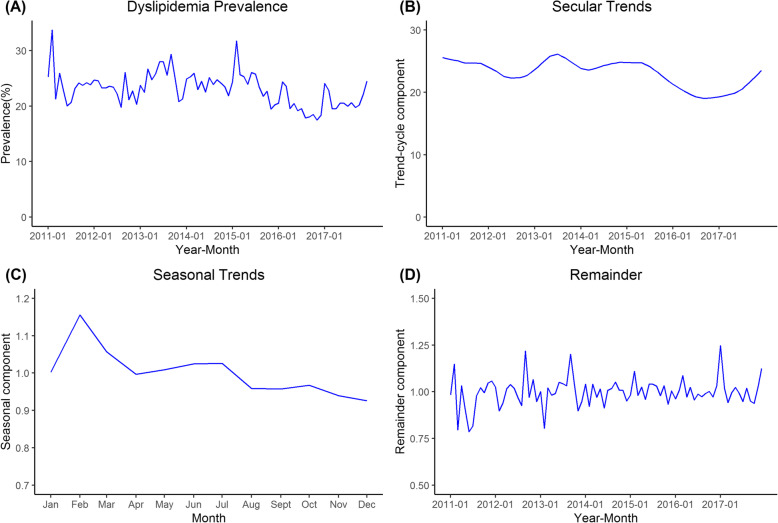
Time series of dyslipidemia prevalence, secular trend component, seasonal component and remainder component by STL decomposition. (A: dyslipidemia prevalence; B: secular trend component; C: seasonal component; D: remainder component)

In Fig. 1(A), significant seasonal variation in dyslipidemia prevalence was observed in the time series during 2011–2017. In Fig. 1(B), the secular trends of dyslipidemia prevalence fluctuated in 2011–2017 with the lowest point in September 2016. A continuous rising trend was observed from September 2016 to December 2017. Figure1(C) shows the seasonal trends of dyslipidemia prevalence over the course of a year. The prevalence from January to March and May to July was above the annual average and λ was equal to 1.00, 1.16, 1.06, 1.01, 1.02, and 1.03. The highest point was observed in February. Figure 1 (D) presents the time series of the remainder of the STL decomposition. Decomposition effects were further assessed by auto-correlation function (ACF) and partial auto-correlation function (PACF) of the remainder. The ACF and PACF of the remainder converged rapidly after 1-month lag (Supplemental Fig. 1). This demonstrated that there was no apparent autocorrelation of the remainder. The Ljung-Box test showed that the time series of the remainder was a white noise series (Ljung-Box test statistic = 0.720, *P* = 0.997). Therefore, time series decomposition in this study achieved a good effect.

### Secular and seasonal trends for the clinical classifications of dyslipidemia

**Fig. 2:**
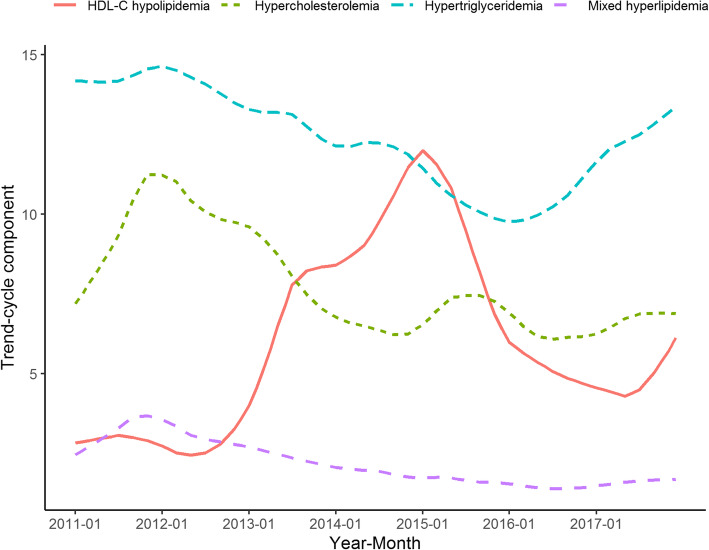
The secular trends of the 4 clinical classifications of dyslipidemia in 2011–2017

In Fig. 2, different secular trends were shown for the 4 clinical classifications. From the middle of 2016 to the end of 2017, a rising trend was observed for the prevalence of hypertriglyceridemia and hypercholesterolemia. A rising trend was also observed for HDL-C hypolipidemia during the second half of 2017.

**Fig. 3:**
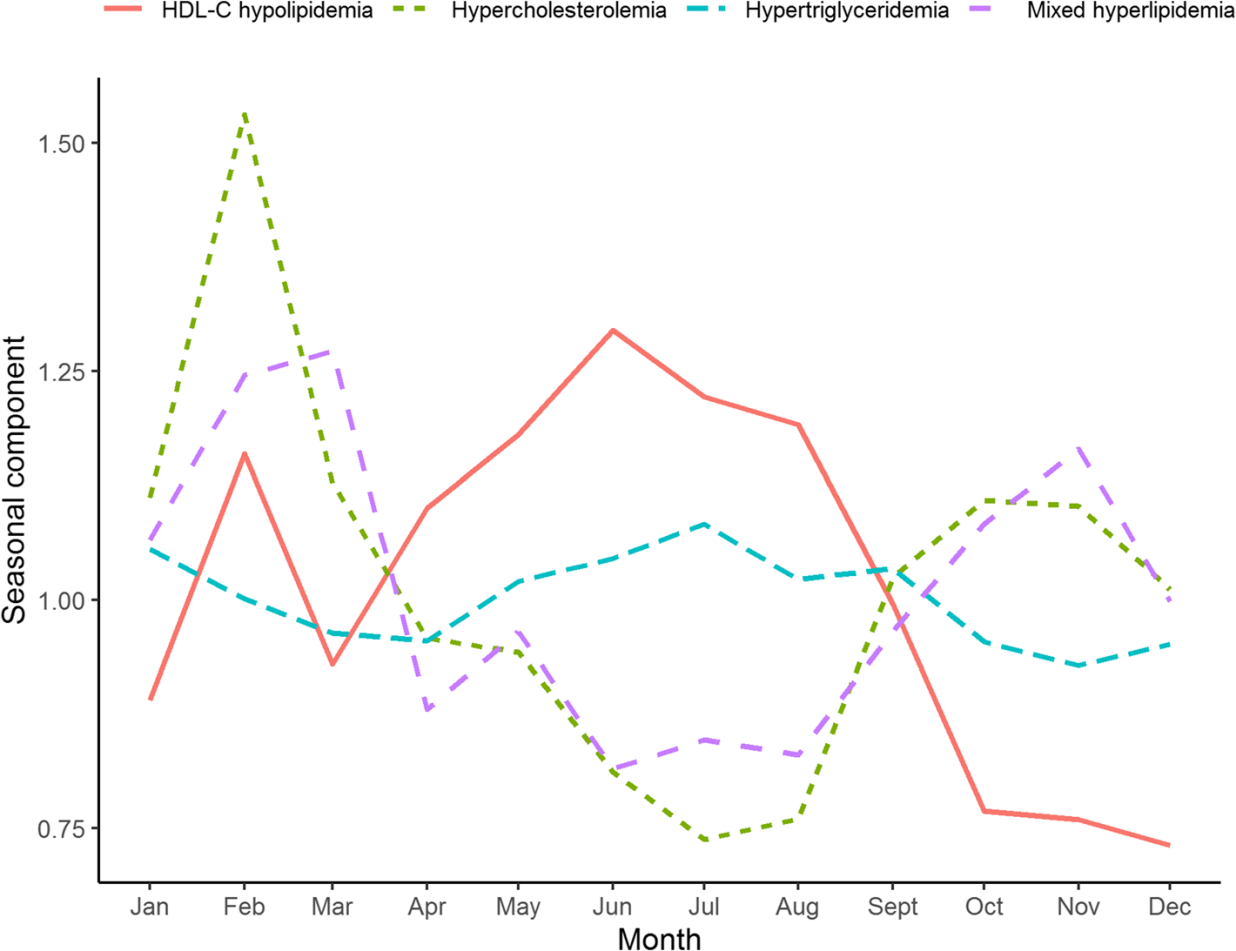
The seasonal trends of the 4 clinical classifications of dyslipidemia

Figure 3 presents the seasonal trends of the 4 clinical classifications. The prevalence of hypercholesterolemia and mixed hyperlipidemia was lower in June to August and higher at the beginning and end of the year. However, the prevalence of HDL-C hypolipidemia showed an increasing trend in spring and summer and a decreasing trend in autumn and winter. The peak hypertriglyceridemia prevalence occurred in July.

### Results of subgroup analysis of dyslipidemia prevalence by sex, age and BMI

**Fig. 4:**
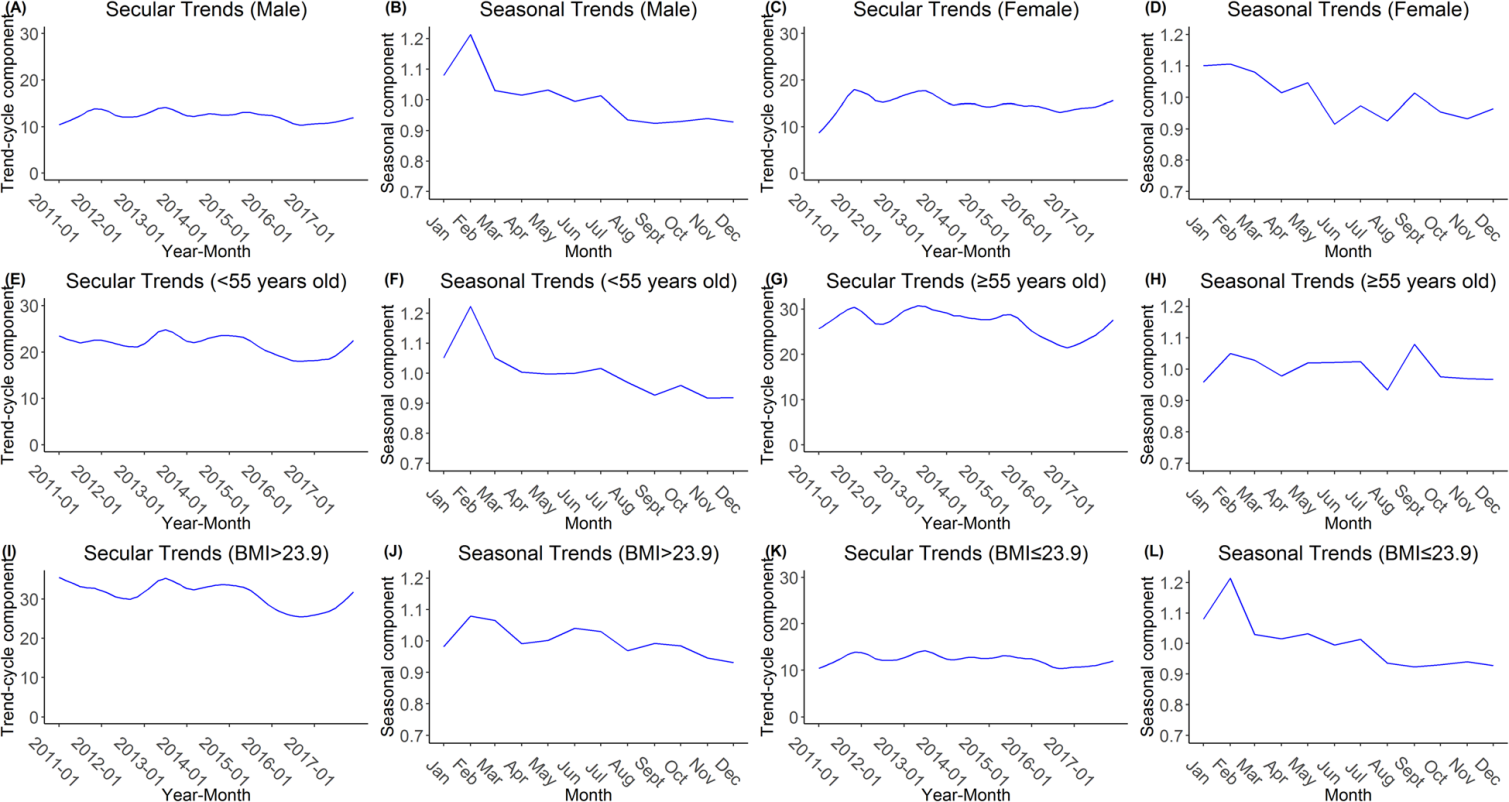
The secular trends and seasonal trends of dyslipidemia prevalence in subgroups by STL decomposition

Figure 4 presents the secular and seasonal trends by STL decomposition in each subgroup. All secular trends of dyslipidemia prevalence among the six subgroups fluctuated in 2011–2017 with a continuous increasing trend from the middle of 2016 to December 2017. Compared to females, a higher point was observed among males in February. Similar trends were also observed among participants aged < 55 years (vs. ≥ 55 years) and participants with BMI ≤ 23.9 (vs. BMI > 23.9).

### Results of sensitivity analysis

In STL decomposition without logarithmic transformation and exponential transformation (additive decomposition), secular and seasonal trends of dyslipidemia prevalence slightly changed but were still in line with multiplicative decomposition (Supplemental Fig. 2). The prevalence in January to March and May to July was also higher than the annual average. This result suggested that the results of this study were robust.

## Discussion

This study collected data from a large sample of an urban Chinese population and found that 21.52 % of participants were diagnosed with dyslipidemia and significant secular and seasonal features for dyslipidemia prevalence were observed. Different seasonal trends were also found in the 4 clinical classifications of dyslipidemia. To our knowledge, this was the first study to explore secular and seasonal trends of dyslipidemia prevalence by STL time series decomposition. Compared with traditional methods, this approach provided a clearer understanding of secular and seasonal trends, which might be a significant supplement to the study of dyslipidemia seasonality.

The finding of the prevalence of dyslipidemia was similar to a previous study for adults of 18–69 years old in Shandong Province in 2011 (22.70 %) [17]. However, the result was lower than that of a previous study in nine provinces of China in 2011, which suggested that the dyslipidemia prevalence in adults was 39.91 % [18]. The same phenomenon was observed in the prevalence of hypercholesterolemia (7.06 % vs. 9.01 %) and hypertriglyceridemia (11.43 % vs. 27.02 %). The reason for this result might be the different dietary patterns between Shandong and other provinces. Given that Shandong is a developed eastern coastal province in China, the residents in Shandong eat more meat that is rich in saturated fat and seafood [19]. Moreover, dyslipidemia was a serious public health problem in China and globally, and it was a strong risk factor for coronary artery diseases [20, 21]. Early intervention of dyslipidemia will contribute to preventing coronary heart disease and improving the prognosis.

The seasonal trends of dyslipidemia prevalence or serum lipid level had been reported in previous studies. Studies in the UK, Japan, and Brazil obtained similar findings to this study about different levels of serum lipids in the summer and winter [22, 23]. However, it was different from those reported in the Helsinki heart study, which showed a drop in HDL-C levels (not HDL-C hypolipidemia prevalence) during mid-winter [24]. The differences in participants’ genetic background, latitude and climate may be the reason for the different results.

For the clinical classifications, the seasonal pattern of hypercholesterolemia and mixed hyperlipidemia showed a decreasing trend from June to August and an increasing trend in the beginning and end of the year. This result was supported by a previous study, which suggested that temperature was an important influencing factor for serum lipids and the decreased air temperature could cause increased plasma lipid levels [25]. Exposure to cold outdoor temperatures increased the basic metabolism, improved the brown adipose tissue activity, and increased serum lipid levels [26]. A peak of hypertriglyceridemia prevalence was found in the summer, which was similar to a study in Poland [26]. A possible explanation is that increased alcohol intake in the summer could increase TG levels [27]. Evidence in previous research suggested that, due to the hot weather, summer was a high season of beer consumption in China [28].

The present study also indicated that the prevalence of dyslipidemia and hypercholesterolemia peaked in February. Generally, the Chinese population observes the most solemn and widely celebrated Spring Festival in February. During the Spring Festival, people like to stay at home and have dinner with family and friends. This may last for one or two weeks. Rich foods and lack of physical exercise for a relatively long time may lead to an increased prevalence of dyslipidemia [29].

## Study strengths and limitations

One advantage of this study is that a large sample size with a relatively long study period was used to explore the secular and seasonal trends of dyslipidemia prevalence. Moreover, a stable and novel time series decomposition method, STL decomposition, was used to separate the secular trend and remainder from the seasonal trend of dyslipidemia.

The limitation of this study must be acknowledged. Despite controlling for the influence of age, sex, and BMI on dyslipidemia prevalence, some influencing factors of dyslipidemia (e.g., lifestyle, diet) were not collected. Secondly, this study is limited to the urban Chinese check-up population included in the routine health check-up system, of which some characteristics were different from those of the general population, rural residents, and people from other countries, which lead to a limited extension of the results to other populations.

## Conclusions

The findings of this study indicated that, after time series decomposition, there were obvious seasonal features for dyslipidemia prevalence. Different seasonal trends were shown in 4 clinical classifications of dyslipidemia. Precautionary measures should be implemented to control the seasonal risks of dyslipidemia. A healthier diet and adequate exercise are needed in specific seasons (e.g., winter) and during holidays. In clinical practice, more seasonal-related guidance should be provided to patients suffering from dyslipidemia or cardiovascular and cerebrovascular diseases. Future research should focus on the mechanism of seasonal or meteorological factors on dyslipidemia and the 4 clinical classifications, including molecular biological mechanisms and changes in behaviors.

## Supplementary Information

**Additional file 1.**

## Data Availability

The data used in the present study are available from the corresponding author on reasonable request.

## Abbreviations

TC: Total cholesterol
TG: Triglyceride
HDL-C: High-density lipoprotein cholesterol
LDL-C: Low-density lipoprotein cholesterol
BMI: Body mass index
STL: Seasonal and trend decomposition using LOESS
CAD: Coronary artery disease
SD: Standard deviation
ACF: Auto-correlation function
PACF: Partial auto-correlation function
