# Supplementary material for: Time series decomposition into dyslipidemia prevalence among urban Chinese population: secular and seasonal trends
Source: Lipids Health Dis. 2021 Sep 22;20:114. doi: 10.1186/s12944-021-01541-6 (PMC8459537; doi:10.1186/s12944-021-01541-6)
Supplement: Supplementary file 1 — Additional file 1. [file 12944_2021_1541_MOESM1_ESM.docx]

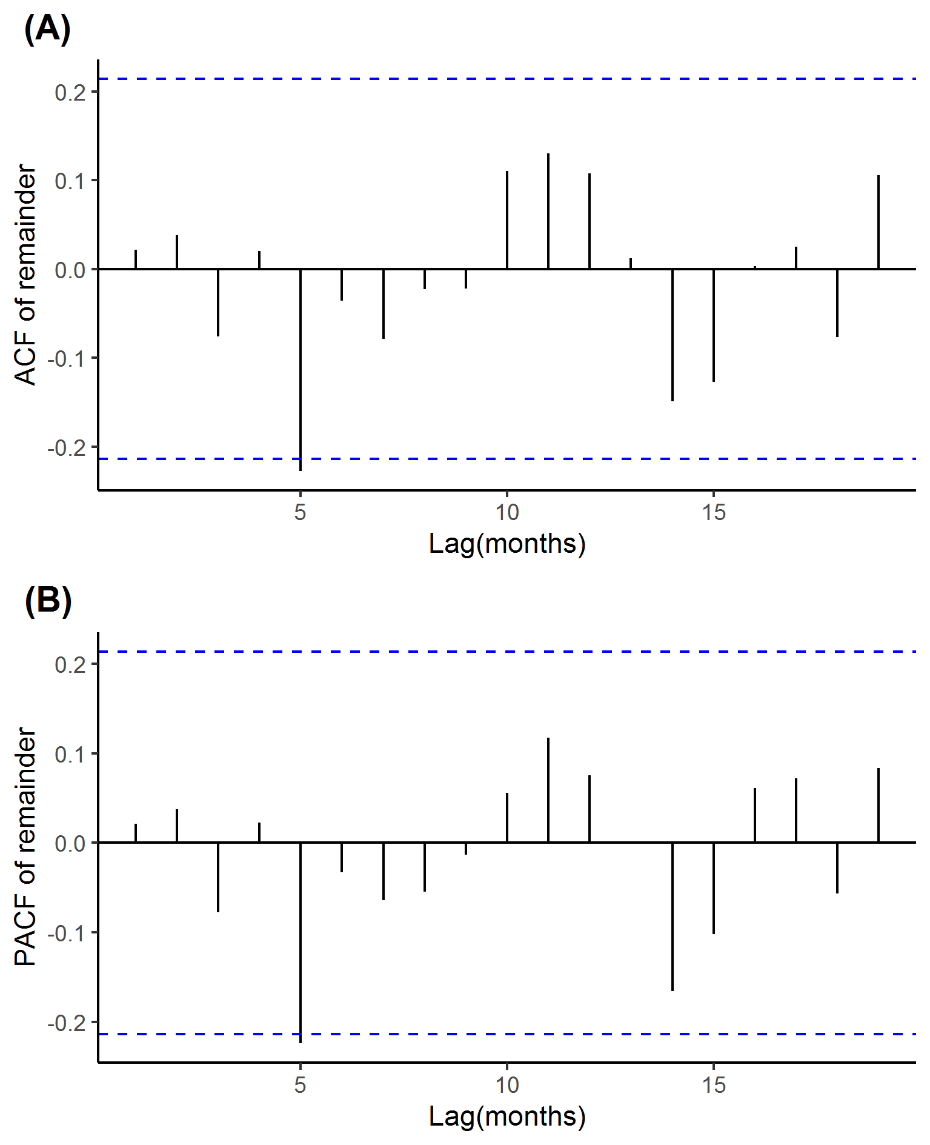


Supplemental Fig.1 ACF (A) and PACF (B) of remainder by STL decomposition.


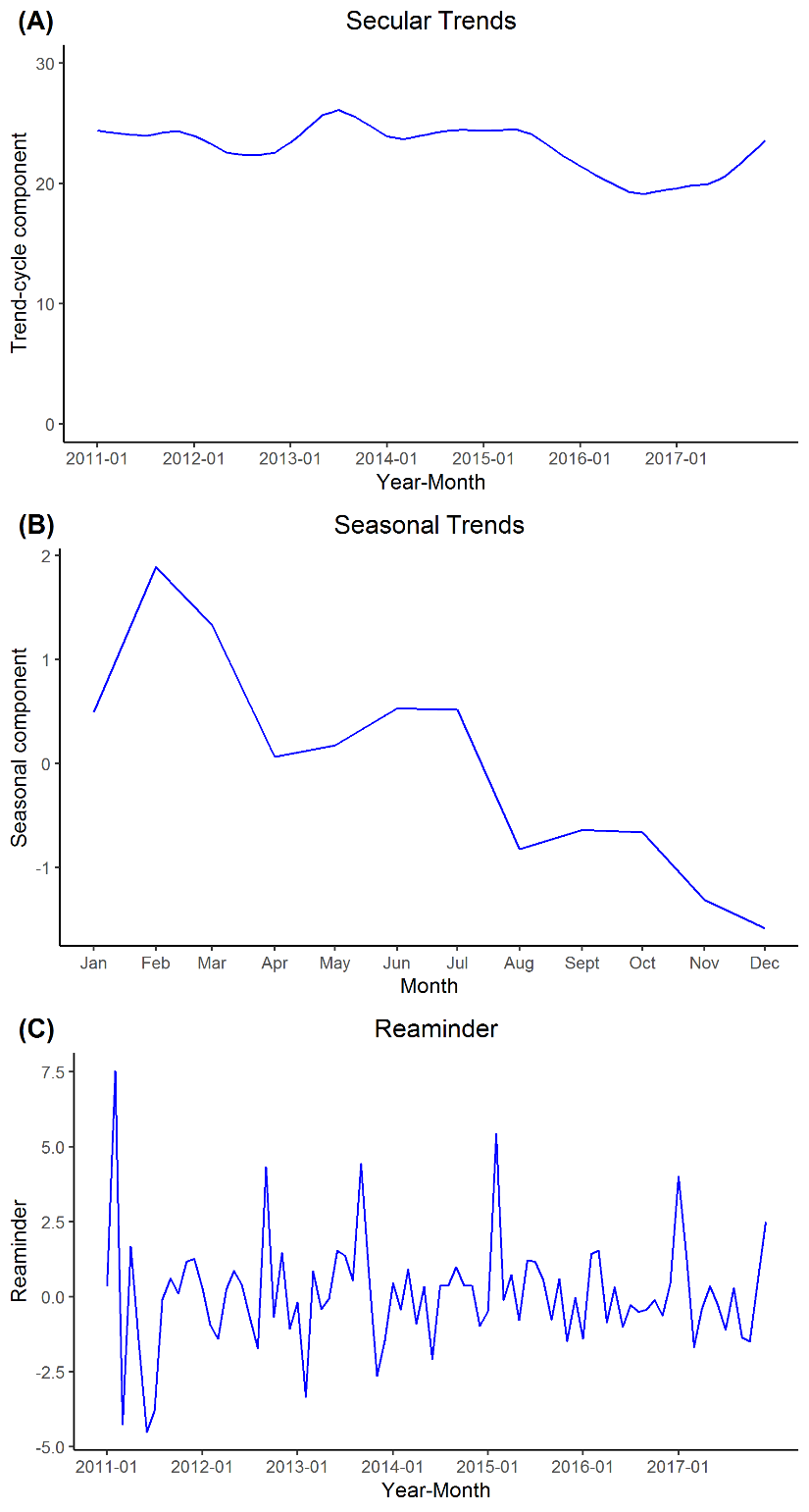


Supplemental Fig.2 The secular trend component, seasonal component and remainder component by STL decomposition without logarithmic transformation and exponential transform. (A: secular trend component; B: seasonal component; C: remainder component)
